# The Summer School Oncology Groningen: Improving a Successful International Course by Refining the Old, Maintaining What’s Good

**DOI:** 10.1007/s13187-020-01944-6

**Published:** 2021-02-01

**Authors:** E. C. E. Boske, P. H. Nienhuis, C. Hammer, M. Jalving, F. A. E. Kruyt, J. de Vries, J. L. N. Roodenburg, M. J. H. Metman, S. Kruijff

**Affiliations:** 1 Faculty of Medical Sciences, University of Groningen, Groningen, The Netherlands; 2 Department of Radiation Oncology, University Medical Center Groningen, University of Groningen, Groningen, The Netherlands; 3 Department of Medical Oncology, University Medical Center Groningen, Groningen, The Netherlands; 4 Department of Surgery, University Medical Center Groningen, Groningen, The Netherlands; 5 Department of Maxillofacial Surgery, University Medical Center Groningen, University of Groningen, Groningen, the Netherlands; 6 Department of Surgical Oncology, University Medical Center Groningen, Groningen, The Netherlands

**Keywords:** Cancer education, Interactive teaching, International network

## Abstract

For more than two decades, the International Summer School Oncology for Medical Students (ISOMS) has organized a biennial 2-week international summer school program in Groningen, the Netherlands. The summer school aims to increase knowledge about general cancer care, reduce fear of talking to cancer patients, and expose students to cancer-related problems. After 22 years, there was a need to improve the summer school format, the application procedure, and the intensity of the course. Here, we describe and evaluate these and additional changes that were made to the program. Several changes were made to the summer school format. The course was shortened from 10 days to a more intensive 7 days. The scientific program was integrated with the clinical program and students were taught scientific writing and presentation skills. The application process involved a personal video pitch. Importantly, the new summer school format was organized by a committee in which medical students had the lead. To evaluate the changes to the summer school, we conducted knowledge tests and regularly obtained feedback. There was a high overall student satisfaction, with a median score of a 9 out of 10. Students appreciated the interactive sessions and practicals and the scientific program, and were satisfied with the course level. All students had improved test scores. Improvement points highlighted the need for a less packed schedule and more lectures on basic oncology principles, or were related to specific lectures. The student-led innovation and adaptation of the ISOMS has been successful.

## Introduction

**Fig. 1:**
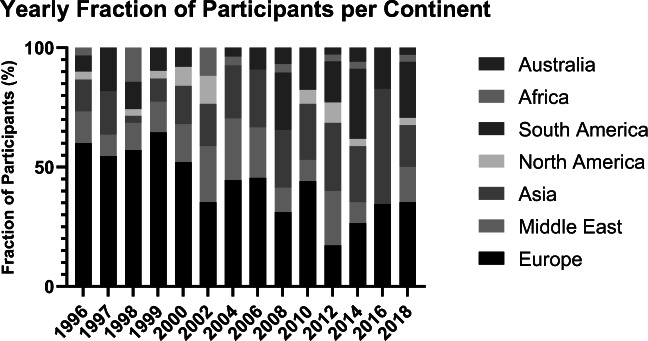
Percentage participant nationalities of all ISOMS editions

Cancer continues to be a significant burden on the international healthcare system and the population’s quality of life. The International Agency for Research on Cancer (IARC) estimates that one-in-five men and one-in-six women will develop cancer over the course of their lifetime worldwide, and that one-in-eight men and one-in-eleven women will die from their disease. Therefore, ongoing education for medical students around the world about oncology remains of paramount importance. The International Summer School Oncology for Medical Students (ISOMS) has been conducted since 1996 at the University Medical Center Groningen (UMCG), The Netherlands. In 1996, the summer school was founded in response to a growing need in cancer care [1]. Back then, studies showed a lack of basic knowledge about principles of cancer care especially in general health practice [2]. Importantly, oncology education could not be found clearly in most curricula in medical schools around the world. For more than two decades, the ISOMS has organized a biennial 2-week summer program aiming to increase knowledge of medical students from all over the world, to reduce fear for cancer, and to create a solid educational program by exposing the students to cancer-related problems. Additionally, in 1999, the Medical University of Vienna started organizing a summer school oncology leading to a strong collaboration organizing the schools in alternating years in two different locations. In the last two decades, the ISOMS organized 14 summer schools in Groningen with a total of 499 students participating from 67 different countries (see Fig. 1). The top 3 countries are Israel (49), Brazil (46), and India (28).

Throughout the years, the program distinguished itself from other oncology summer schools by its highly interactive style of education, multidisciplinary aspects, and attention for social context. In previous years, evaluations have proved ISOMS to be highly appreciated by students. Students not only benefitted from the multidisciplinary approach, the patient encounters, the psychosocial concern, poster presentations, and the quality of teaching but also from meeting medical students from all over the world with an interest in oncology. This led to a tight future professional network. Next to this, the low threshold for contact between teachers, students, and patients has been extremely appreciated and created a safe learning environment to talk about difficult topics such as end of life and palliative management.

Despite these positive aspects, there was a need for change to the summer school program. Previously, students could choose between a science and clinically oriented track. Feedback on previous summer schools showed us demand for a format in which both scientific as well as clinical aspects were integrated. Next to this, the application and selection process of the students needed to be altered. In the past, students submitted their final abstract and the summer school provided no opportunity to improve the abstract. Most teachers of the summer school are medical doctors, professors, and researchers. A 10-day program is too intensive for both students and teachers. A 10-day program close to the summer vacation is too demanding for the medical staff because of less staff availability and a higher caseload.

After 22 years, we refined ISOMS by building further on previous success. We innovated the school by making several alterations. In this manuscript, we describe the measures taken in the summer of 2018 and how these were evaluated by the students and what lessons it teaches us for future summer schools.

## Methods

The new course format was designed by a medical student committee, supported by a committee of doctors and researchers. To rigorously evaluate the new organization and program, several methods were used to assess the educational effect and satisfaction of the participating students. Students were asked to fill in evaluation forms at the end of each day. These evaluation forms regarded grades and written feedback for all educational sessions that day. This allows us to pinpoint powerful points and improvement points for future summer schools. Additionally, students were obliged to fill in an extensive evaluation form on the last day of the summer school, consisting of 58 questions. All evaluation forms were completed anonymously. To assess the efficacy of the promotion, students were asked to fill in via which medium they heard about the existence of the summer school. Satisfaction with the application process was evaluated in an anonymous survey among all students. We attempted to objectify the educational yield using knowledge test scores from before and after the summer school. We described noteworthy alterations to and important aspects of the summer school organization below.

### Promotion

To promote the ISOMS and reach highly qualified and motivated medical students, several mediums were used. Via the University of Groningen, partner universities all around the world were sent posters and information on the summer school. Additionally, information was published on both the University of Groningen website and on the ISOMS’ own website. The summer school was also indexed on summerschoolsineurope.eu, which is a central website where students can find information about many summer schools. Importantly, we created a Facebook page and used paid Facebook promotion, this way we could reach more interested students and keep in contact with each other. As part of the application process, students were asked to answer the question, by which means they had initially learnt to know the summer school.

### Application Process

The application process was originally based on a letter of recommendation, a resume, and a scientific abstract. Students were accepted for attending the summer school if the abstract was of an acceptable quality by reviewing and after payment had been fulfilled. This process was very intensive and rather complicated for logistic reasons. The abstracts were sent back and forth multiple times to bring it to the right levels of standard. Therefore, now the abstract was allowed to be a rough first version as the students were challenged in a later phase to improve this abstract during the summer school under guidance. Throughout the years, ISOMS received many applications of qualified and motivated students who did not have the means to do research and write an abstract. Therefore, in the new version, students were asked to send in a clinical case abstract if they were not capable of writing a scientific abstract before the application deadline. The case illustrates an important oncological concept or treatment plan based on the currently available evidence. To strike the interactive and social approach of ISOMS, we introduced another element to the application procedure. Students were asked to record a personal 1-min introduction video pitching their motivation. If for technical reasons they were not able to record a video, the option was given to write a motivational letter.

### Educational Program

The summer school was shortened to an intense 7-day program, with a break of 2 days in between. The goal was to develop a more interactive and omnifarious educational program. A good example of this is the live multidisciplinary meeting (MDM) that was based on role-playing by the students acting as specialists in an MDM with real medical cases. Another example is the plenary clinical reasoning session in which the gained knowledge and skills of the students were challenged. In this educational session, students were confronted with a clinical case and had to ask for information and diagnostics to solve it and coming up with a differential diagnosis in the end.

We aimed for a diverse and broad program. Whilst in the past the program was split into two tracks, the new program of 2018 entailed both clinical and research aspects for all students. An example is the research-themed day in which the new subjects, such as the European Society for Medical Oncology (ESMO) clinical evidence scale and quality-adjusted life years, the use of intraoperative imaging, proton therapy, and a visit to the Groningen Proton Therapy Centre, were included. Other new subjects were public speaking skills, practical sessions on pathology and anatomy, and a focus on the quality of life after cancer of the so-called cancer survivors. To accommodate all the educational activities, our educational program featured teaching on the most common cancer types. Therefore, lectures on esophageal, hematological, hepato-pancreato-biliary, pediatric, and brain cancers were removed from the program.

To objectively assess the educational yield of ISOMS, students were obliged to make a knowledge test on the first and last day of the summer school. This test consisted of 51 multiple-choice questions. The questions were made by the teachers of the summer school. The order of the questions was randomized for each student. After finishing the test, the student would only see the percentage of correct answers given.

### Scientific Program

The scientific program was renewed to an interactive learning format working in small groups. The group of students was divided into six groups of five to six students, each guided by experienced scientific mentors.

In two sessions of 90 min, students were taught how to improve their scientific writing and presenting skills. In the first meeting, students and their mentors got acquainted and students shortly presented their abstract of clinical case. Next, the mentor plenarily gave students advice on the structure and format of a scientific abstract and of a clinical case. In the remaining part of the session, students had the opportunity to improve their abstract or clinical case and send it to their mentor. Before the second meeting, students had received feedback from their mentors and prepared a presentation based on the improved abstract or clinical case. During the second meeting, all students presented their improved work in front of their group and mentor. In this session, students received feedback from their mentors and their peers. The taught presentation format was similar to that of an oral presentation at a scientific congress, with 10 min of presenting and 5 min of discussion and feedback. Lastly, the supervisor—together with the group of students—nominated one student with the best abstract to present on the last day of ISOMS for the whole group, competing for the Summer School Scientific prize. The best abstract or clinical case presentation was chosen by a jury of experienced researchers and clinicians.

### Social Program

One of the aims of the summer school is to create a social environment for a future professional network. This involves not only relations between students but also relations between students and teachers involved in the summer school program. In the new format, we introduced new elements to the program to lower the threshold created by hierarchy and facilitate informal social interaction between students and professors. We organized a dinner at a doctor’s home for all the students and a few medical doctors. In addition, teachers were invited for all lunches during the educational program. Lastly, we invited the staff and several teachers to attend some dinners to mingle with the students.

## Results

### Overall Evaluation of the Summer School

**Fig. 2:**
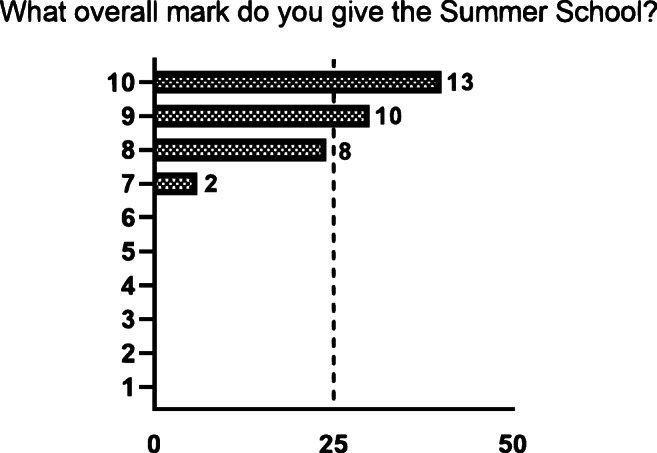
A bar graph showing the distribution of overall marks given by students on a scale of 1 to 10, with 1 being very band and 10 being excellent. The *X*-axis shows the percentage per mark. The number behind each bar represents the actual number of students in that category

**Fig. 3:**
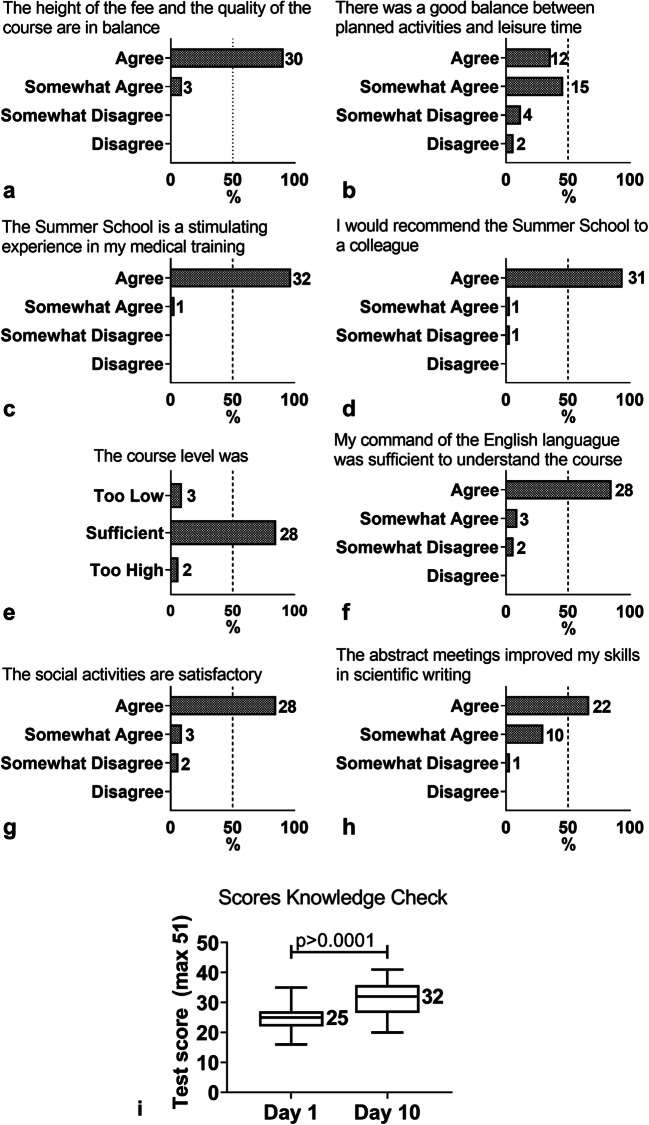
**a**–**h** Bar graphs showing the responses of students to several aspects of the Summer School and **i** boxplots showing the scores obtained from the knowledge check. Overall aspects (**a**–**d**), the educational program (**e** and **f**), the social program (**g**), and the scientific program (**h**). The *X*-axis shows the percentage of students per response and the number behind each bar represents the actual number of students per response. After conducting the knowledge test (**i**), the median score on day 1 and day 10 differed significantly (*p* < 0.0001). The *Y*-axis represents the median score

There was a high overall student satisfaction, with a median score of 9 out of 10 (Fig. 2). Moreover, students found the summer school a worthwhile investment in their medical training and would recommend it to their colleagues (Fig. 3c and d). Importantly, multiple students found that there were too many planned activities and would have appreciated more leisure time (Fig. 3b).

### Promotion

Out of 32 students, 8 learned about the ISOMS via another student that had participated in the summer school before. Seven students learned about the summer school via the Internet (other than Facebook), 6 via a doctor or professor, 4 via a partner university, and 3 via Facebook. The remaining 4 students had heard about the summer school via a poster, a Winter School, a friend, and the Medical University of Vienna.

### Application Process

We received 64 notions of interest via e-mail of which 43 included the required application documents (letter of recommendation, resume, clinical case or abstract, and a video or motivational letter). A video motivation was received from 14 applicants and 29 applicants included a letter of motivation. After the selection procedure, we selected 32 students, of which 13 with an abstract and 19 with a clinical case report.

### Educational Program

Overall, the students were satisfied with the educational program. All students (somewhat) agreed that the content of the course was interesting and well presented, and that it gave them better insight and factual knowledge on oncology. Importantly, 85% of students found the level of the course was neither too low nor too high (Fig. 3e). Some students found the level of English used in the course too high (Fig. 3f).

In free-text feedback, students would have liked to see more lectures on the basics of oncology. Regarding to specific subjects, students were disappointed that hemato-oncology and neuro-oncology were missing in this edition of the summer school.

The daily evaluations of the educational program revealed that students regarded the interactive workshops, ward rounds, multidisciplinary meeting simulation, and the clinical reasoning lectures as the best parts of the summer school. All students were satisfied with the interaction with the teachers and felt they had ample opportunity to ask questions. Moreover, the highest-rated educational activities were those that featured patients.

Some parts of the educational program were evaluated with more criticism. Some elements of lectures overlapped with others and two specific lectures did not meet expectations with regard to the content. A common point of criticism from the students concerned the intense schedule. Students mentioned that some days were too long and that there were not enough breaks. Several students would have appreciated more time to individually study.

The mean overall score on the knowledge check increased by 12% (SD 7%) (*p* < 0.0001) on the final day of ISOMS compared to the first day (Fig. 3i). One student did not increase in score, whereas the highest increase was 27%. The range in scores increased from 19 to 21 out of 51 questions. There was no association between the scores of questions and the evaluation of the lecture on the same subject.

### Scientific Program

Overall evaluation of the scientific program revealed that students especially rated the abstract meetings highly. They appreciated the small group setting and the open atmosphere in the group. Most students felt that the meetings improved their scientific writing skills because the teachers provided the students with useful and hands-on skills (Fig. 3f).

Student evaluation revealed that there was not enough time during the abstract meetings. Additionally, the organizing committee experienced that the abstract presentations were stressful for many students.

Evaluation of the lab excursions showed that students missed the opportunity to practice basic lab-techniques.

### Social Program

Overall, students were satisfied with the social program (Fig. 3g). Most students mentioned that they appreciated the opportunity to mingle with their fellow students and with teachers. The international cooking event is frequently mentioned as a highlight of the social program.

The most mentioned improvement point for the social program is the busy schedule; there was too little time between the end of the educational program and the start of the social program.

## Discussion

For more than two decades, the ISOMS has organized a biennial 2-week summer school program aiming to increase knowledge about general cancer care for medical students from all over the world, to reduce fear for cancer, and to create exposure to cancer-related problems. Just a few years after the start in 1999, we initiated a close collaboration with the Medical University of Vienna, which marked the start of our summer schools being organized in alternating years. After 22 years, we refined ISOMS elements but at the same time built further on the successful basis. We innovated the school program by making several alterations. The summer school was shortened, the application process was altered, and the educational program was changed into a more interactive format. The students had daily teaching to improve their scientific skills and new elements were added to the educational program. A feedback evaluation and knowledge pre- and post-test illustrated an increase in knowledge. We may conclude that the innovation of the educational monument of the ISOMS in Groningen has been a success.

Worldwide cancer education curricula for medical students are often still very heterogeneous and lack a solid basis [3–6]. No common curriculum can be identified among Western medical schools containing basic elements of multi- and interdisciplinary approach of oncology teaching [7]. Historically, back in the 1990s, this deficit created the need for an international, integrated, multidisciplinary oncology school for medical students, leading to the establishment of the ISOMS [2, 6, 8–10]. Today, the high number of applications by our medical students and summer school candidates, as well as the lack of solid, protocolled cancer education in many university curricula, illustrates there is still a solid need for added education of oncology topics for medical students [7].

As the worldwide incidence of cancer is still increasing rapidly [11], it remains crucial to create well established educational platforms in which multidisciplinary-focused oncology education is offered. This holds true for medical students that are interested in oncology but also for future professionals who will have to deal with the cancer in various medical roles in soon. In other words, not only the future oncologist will deal with cancer patients but also the doctors that are involved in general health practice. Such are general practitioners, who often lack good and high quality oncology training [3–6].

Continuously changing oncology treatment protocols and ongoing evolving oncology science added by the complexity of multidisciplinary approaches make the design and execution of a solid educational oncology curriculum challenging. Anno 2020, the teaching of this dynamic discipline cannot only be offered in books and must cover a variety of aspects. It should include a basic knowledge about the classic pillars of cancer treatments such as oncologic surgery, medical oncology, and radiotherapy, combined with an understanding of translational science, the development of new diagnostic modalities, and basic epidemiology. More importantly, well-developed communications skills and the basic elements of palliative care should be a vast element of the curriculum. A universal rule is that without well-developed communication skills, a professional can never gain the trust of their patients who are going through a dramatic episode of their lives.

By offering a solid curriculum with decades of experience and knowledge transfer from teacher to student, the ISOMS provides an educational platform for every medical student around the world. Despite this, the surrounding changes, such as digitalization and the needs of the students themselves, demanded an adaptation of the educational infrastructure. Therefore, we innovated the summer school without changing its basis and philosophy; an ongoing spread of oncology knowledge for medical students.

Although the WHO advises 10 days of oncology education, we decided to reduce the educational schedule to 7 days by making the program more intensive while maintaining the range of topics [2, 9]. For the participants as well as the teachers and faculty, a 7-day length seemed more realistic because it increased availability. Also, for the application process, we added a personal 1-min film to disclose the participants’ motivation. This led to a more personal idea about candidates and what their primary drives were to attend the summer school. An impressive resume can sometimes disguise a thin motivation. Since the summer school basis is defined by a highly interactive and social character, an introductory film helped to select students better based on their motivation.

The scientific-educational program was renewed to a more interactive format that focused on working in small groups. Under the supervision of an experienced researcher, the students learned how to write a scientific abstract or clinical case. This proved to be one of the summer school’s best-rated educational activities. However, the level of scientific knowledge of the students varied greatly, as did the quality of the abstracts and clinical cases. Therefore, future ISOMS editions will work with scientific assignments where the students will have to write a research proposal. The small group setting will remain crucial in this new format. Importantly, these scientific meetings will be expanded to 3 h. This serves the purpose of increasing the quality of education as well as a means to decrease stress levels for students, who now need less time working on their presentations after the educational program ended. Additionally, this extra time may also be used to improve the quality of peer-to-peer feedback, making use of standardized formats such as the Pendleton rules [12].

We also added a role-playing element for the students of a multidisciplinary meeting. Clinical decision-making in oncology is based on interdisciplinary team communication and application of treatment concepts and algorithms in a multidisciplinary setting. This requires a high level of fundamental pre-clinical and clinical knowledge and the ability to apply the acquired knowledge tailored to a specific patient. This ISOMS edition included a simulated multidisciplinary team (MDT) meeting, which was well-received. The simulated MDT meeting involved many different specialists—as does a real MDT meeting—and therefore required strict organization, both in terms of time management and instructions. In future ISOMS editions, the framework for this session will remain the same. We may allocate extra time to ensure that students have the time to prepare the patient cases.

Finally, we tested students’ knowledge using two “knowledge checks,” at day 1 and day 7 of the summer school. Although evaluation results revealed that students felt that ISOMS had contributed to their factual knowledge and understanding of oncology, the knowledge checks provide objective measures of knowledge gain. All students had increased scores on the knowledge check, with a mean increase of 12% in their test scores. The spread of scores in terms of range also increased, showing that some students benefitted more from the summer school than others in terms of knowledge gain. However, it must be noted that this way of objective assessment of students’ knowledge gain must be interpreted with caution. First, students received the same knowledge check twice, meaning that they may have remembered certain questions rather than increased their overall understanding of oncology. Second, not all educational activities were equally represented in questions in the knowledge check. Therefore, the measured knowledge gain does not represent all the subjects taught in the summer school. In the next ISOMS edition, we aim to greatly revise and improve our objective measurement of knowledge gain. First, we will increase the number of questions by making a question bank. In the knowledge check, the number of questions per subject will be determined by the number of hours of teaching dedicated to this subject. This way, the knowledge check will be a more honest reflection of the summer school’s curriculum. Second, we will make two separate knowledge checks, containing different questions. On the first day, we will divide all students into two groups. Each group receives one version of the knowledge check. On the last day, the same groups will receive the other version of the knowledge check. We hereby subvert the potential bias of students remembering certain questions as well as ensure the validity of the tests. This specific way of testing has been performed before in the summer school, as described by De Vries et al [2]. Last, we aim to use the knowledge check on day 1 to give feedback to students based on their results. Based on the results from the first knowledge check, we can give learning objectives to individual students. These learning objectives will then allow the student to pay extra attention to the specific subjects taught during the summer school that he or she finds difficult.

Interestingly, we did not find any relationship between the appreciation of a lecture and increased knowledge gain on the same subject. This shows that, in this setting, highly rated educational activities may not increase the transfer of knowledge. However, because of the biases described above, this must be interpreted with caution. The participating students represented a selection of students with a special interest in oncology and were highly motivated to learn more about this topic. Also, their gratefulness for the effort invested by staff and organizing students might have made them less critical in their judgments.

There is no doubt that the initiatives of summer schools worldwide are still needed and fruitful to add oncological knowledge to our future oncology physicians. While the increase of cancer in low- and middle-income countries is exploding, our staff is considering organizing an ISOMS on a different location in for instance sub-Saharan Africa such as Malawi [13], just as was done before successfully in the winter school in India [9].

In conclusion, we want to emphasize the need of spreading oncological knowledge, especially to underserved communities. The ISOMS aims to allow students from all over the world to improve their understanding, increase their scientific skills, and learn from other students. The ISOMS 2018 was highly rated and is recommended by students. The summer school provided a stimulating learning environment and increased students’ knowledge on oncology. The majority of the alterations to the summer school program were received well by students and provided the basis for ongoing innovation and success of this summer school. The basis for oncology taught in the ISOMS will lay the foundation for these future doctors to serve cancer patients.

## Data Availability

Not applicable.
